# Suppression of liver Apo E secretion leads to HDL/cholesterol immaturity in rats administered ethinylestradiol

**DOI:** 10.1002/2211-5463.12098

**Published:** 2016-07-21

**Authors:** Kosuke Yamaguchi, Mariko Ishii, Naoyuki Maeda, Hidetomo Iwano, Hiroshi Yokota

**Affiliations:** ^1^ Laboratory of Veterinary BiochemistrySchool of Veterinary MedicineRakuno Gakuken UniversityEbetsuJapan; ^2^ Safety Research Institute for Chemical Compounds Co. LtdSapporoJapan

**Keywords:** Apo E, cholesterol, diethylstilbestrol, endocrine disruptor, ethinylestradiol, HDL

## Abstract

Ethinylestradiol (EE), a main component of the combined oral contraceptive pill, is associated with an increased risk of arterial diseases. However, the toxicity mechanism of EE is poorly understood. In this study, we found that the exposure to EE reduced the serum apolipoprotein E (Apo E) level and high‐density lipoprotein (HDL)/cholesterol concentration in adult female rats. Diethylstilbestrol showed the same effects and both reductions were suppressed by coadministration of tamoxifen (TAM). Liver perfusion experiments revealed that the secretion rate of Apo E from the liver was significantly reduced. It is concluded that EE damages the maturation of HDL/cholesterol by delaying Apo E secretion from the liver, and this may lead to an increased risk of arterial diseases, such as atheromas.

**Abbreviations**
Apo A1: apolipoprotein A1
Apo E: apolipoprotein E
BPB: bromophenol blue
COCs: combined oral contraceptive
DES: diethylstilbestrol
DTT: dithiothreitol
ECL: electrochemical luminescence
EDCs: endocrine‐disrupting chemicals
EE: ethinylestradiol
ER: estrogen receptor
HDL: high‐density lipoprotein
LC‐MS: liquid chromatography‐mass spectrometry
LDL: low‐density lipoprotein
LDLR: low‐density lipoprotein receptor
LXR: liver X‐receptor
MALDI‐TOF MS: matrix‐associated laser desorption ionization‐time‐of‐flight mass spectrometry
PVDF: polyvinylidene fluoride
TAM: tamoxifen
VLDL: very low‐density lipoprotein
VTE: venous thrombosis events

Endocrine‐disrupting chemicals (EDCs) can mimic, interfere or block the function of hormones and can also act as agonists and/or antagonists of estrogen and androgen receptors. EDCs are linked to birth defects, decreased fertility, unbalanced sex hormones, defects of the immune system, and neuronal adverse effects 1, 2, 3. The most investigated endocrine disruptor, diethylstilbestrol (DES), is a synthetic nonsteroidal estrogen used in the United States and Europe from the mid‐1940s to the 1970s for the prevention of spontaneous abortion and premature delivery. DES causes severe endocrine toxicities in human 4, 5, 6, 7, 8, 9, 10; however, the detailed mechanisms of action of DES are not well understood. DES has also been used for prostate cancer therapy in Europe after failure with initial hormonal therapy 11. A higher dose of DES‐phosphate (500–1000 mg·day^−1^ for 2–5 weeks) suppresses the serum levels of testosterone for the treatment of prostate cancer patients 12. For the prevention of harmful repeated incidents, we must identify the actual disruptors of various chemicals to understand the toxicological mechanisms of endocrine disruption processes of each disruptor. Using *in vivo* assays with experimental animals, it is difficult to screen for the endocrine disruption activity of various chemicals because the toxicological processes *in vivo* are complex and time consuming to obtain the final assessments. We use *in vitro* assay systems, including hormone receptor binding assays, cell proliferation assays, receptor gene assays in yeast or mammalian cells, and an estrogen sensitive gene assay in cell lines 13 to evaluate the endocrine‐disrupting activity of chemical compounds. However, it is difficult to investigate the mechanisms underlying endocrine disruption *in vitro*. These simple methods may be used to assess the direct adverse effects on target organs, although the indirect effects of chemicals after metabolism in other organs were not evaluated. Furthermore, other endocrine disruption processes caused by defects in endocrine regulation resulting from the association between endocrine organs and extra‐endocrine organs could not be evaluated using these assays.

Our laboratory recently identified the indirect adverse effects of diethylstilbestrol (DES) on adrenal steroidogenesis 14. We showed that DES caused a decrease in serum high‐density lipoprotein (HDL) cholesterol, the main source of adrenal steroidogenesis, due to the inhibition of apolipoprotein E (Apo E) secretion from the liver 15. This is the first report about indirect adverse effects on the target organs of endocrine disruptors.

The association between thrombosis and combined oral contraceptives (COCs) containing estrogen and progestin was reported soon after their widespread use 16. The risks of venous thrombosis events (VTEs) with COCs are associated with the factor V Leiden mutation 17. Estrogens in contraceptives cause changes in the plasma coagulation profiles, resulting in an increased risk for VTEs 18. 17α‐Ethinylestradiol (EE)‐containing patches may lead to an increased risk of VTEs compared to estradiol‐containing patches 19, which do not affect the risk of VTEs 20. Recently, EE altered the transcript levels of coagulation genes via the estrogen receptor a (ERa) 21. These results indicate that EE is closely related to the increased risk of VTEs in women using COCs.

In this study, we found that the adverse effects of DES occurred after binding to ER and a synthetic estrogen, EE, also suppressed the HDL cholesterol levels having a crucial role in atherosclerosis.

## Materials and methods

### Materials

DES, EE, and tamoxifen (TAM) were purchased from the Sigma‐Aldrich (St. Louis, MO, USA). Olive oil was obtained from Nakarai Yakuhin Co (Kyoto, Japan).

### Treatment of animals

Male or female Sprague–Dawley rats (weight, 280 ± 20 g and age 9–10 weeks) were fed, housed, and allowed to adapt to their environments for 1 week before the experiments. The rats received an oral dose of 0.1 mg DES, EE, or both DES and TAM dissolved in 0.5 mL of olive oil (~ 330 μg DES, EE or both per kg of body weight). Control rats received 0.5 mL of olive oil only.

### Sample preparation from administered rats

Blood was collected from the abdominal aorta of the animals via exsanguination under pentobarbital anesthesia. After dissection, the organs were excised post‐mortem, weighed and then immediately frozen and stored at −25 °C. The blood was centrifuged at 900 ***g*** for 20 min at 4 °C after 30 min on ice. The supernatant was used as the serum from the rats. The liver tissue (1 g) was gently homogenized in a 0.25 m sucrose solution (4 mL) at 4 °C and centrifuged at 900 ***g*** for 10 min at 4 °C for the removal of nuclei fraction and undisrupted cells and tissues. The supernatant was centrifuged at 8000 ***g*** for 10 min at 4 °C for the removal of the mitochondrial fraction. The final supernatant was used as the cell homogenates containing the microsomal and cytosol fractions. All animals were treated according to the Laboratory Animal Control Guidelines of Rakuno Gakuen University, which conform to Guide for the Care and Use of Laboratory Animals of the National Institutes of Health, USA.

### Liver perfusion

The surgical procedures and the liver perfusion were performed as previously described 15. The livers of the control and EE‐exposed rats were isolated and liver perfusion was performed with 300 mL perfusate as previously described 15. The perfusate was consisted of Krebs–Henseleit buffer (95%), FBS (5%), glucose (10 mm), and HEPES (10 mm). The perfusate was incubated at 37 °C and oxygenated with 95% O_2_ and 5% CO_2_. We collected 1 mL perfusate every 10 min and then immediately froze and stored the samples at −25 °C for western blotting analysis.

### Western blotting analysis

The protein concentration of the microsomal fraction was estimated by Lowry protein assay. For the detection of Apo E, apolipoprotein A1 (Apo A1) and β‐actin, the microsomal fraction and serum were separated via SDS/PAGE and then transferred to polyvinylidene fluoride (PVDF) membranes (ATTO, Tokyo, Japan) for western blot analysis. The membranes were probed with the following primary antibodies as previously described: rabbit polyclonal antibodies against rat Apo E, Apo A1, and β‐actin. The expression levels of each protein were analyzed densitometrically using the cs analyzer ver 3.0 (ATTO).

### Preparation of HDL and LDL/VLDL fractions

Separation of HDL and low‐density lipoprotein (LDL)/ very low‐density lipoprotein (VLDL) was performed as described in the protocol provided with the HDL and LDL/VLDL Quantification Kit (BioVision, Milpitas, CA, USA). Serum samples (100 μL) were combined with 2× Precipitate buffer (100 μL) and incubated for 10 min at room temperature. The solution was centrifuged at 2000 ***g*** for 10 min. The supernatant was retained as the HDL fraction. The precipitate was recentrifuged, and trace amounts of the HDL supernatant were carefully removed. The precipitate, which is the LDL/VLDL fraction, was resuspended in 200 μL PBS.

### Cholesterol analysis

The total cholesterol level in the serum was assayed using the Cholesterol E Kit (Wako, Osaka, Japan) with cholesterol oxidase and cholesterol esterase. Free cholesterol was determined using the same procedure without cholesterol esterase. The cholesterol ester level was calculated using the total and free cholesterol levels. The adrenal gland tissue was gently homogenized in 0.25 m sucrose solution (200 μL), followed by centrifugation at 900 ***g*** for 10 min. The amount of total cholesterol in the resulting supernatant was assayed according to the manufacturer's protocol.

### Statistical analysis

Results are expressed as the mean ± SEM of three to five independent experiments. Statistical analysis was performed using the *F*‐test and Student's *t*‐test.

## Results

**Figure 1:**
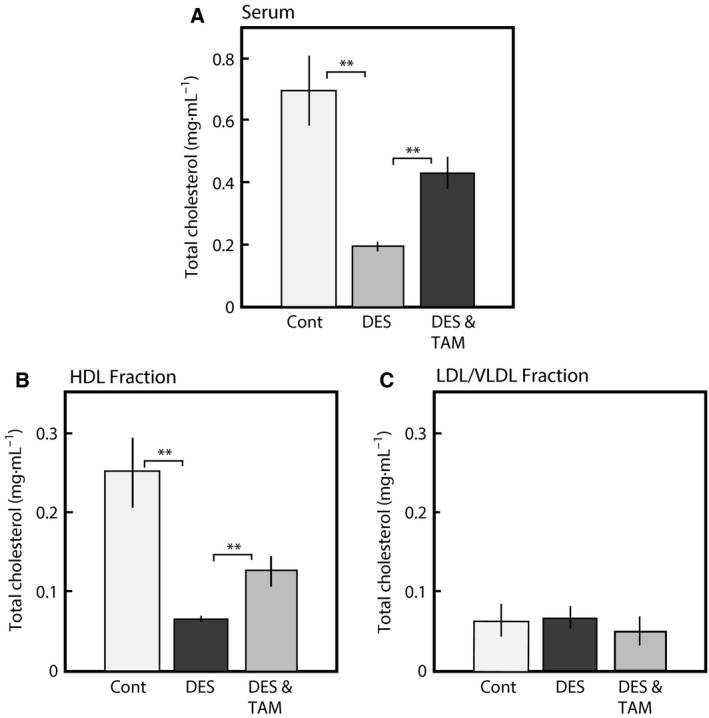
Effects of tamoxifen (TAM) on the cholesterol levels in the adult male rats administered with DES. DES was orally administered for 1 week [0.1 (mg DES per rat) per 2 days] to adult male rats. The blood was collected at 24 h after the final dose as described in the Materials and methods section. Serum HDL and LDL/VLDL fractions were prepared as described in the Materials and methods section. The amount of total cholesterol in the serum (A), HDL fraction (B), and LDL/VLDL fraction (C) of rats administered DES for 1 week (DES), coadministered DES and antiestrogen, tamoxifen (DES and TAM) and of control rats administered olive oil only (Cont) were determined. Cholesterol levels were determined by the enzyme methods using oxidase and cholesterol esterase as described in the Materials and methods section. The data for each group represent the mean ± SEM of three to five rats. **P < 0.01 compared with each group.

**Figure 2:**
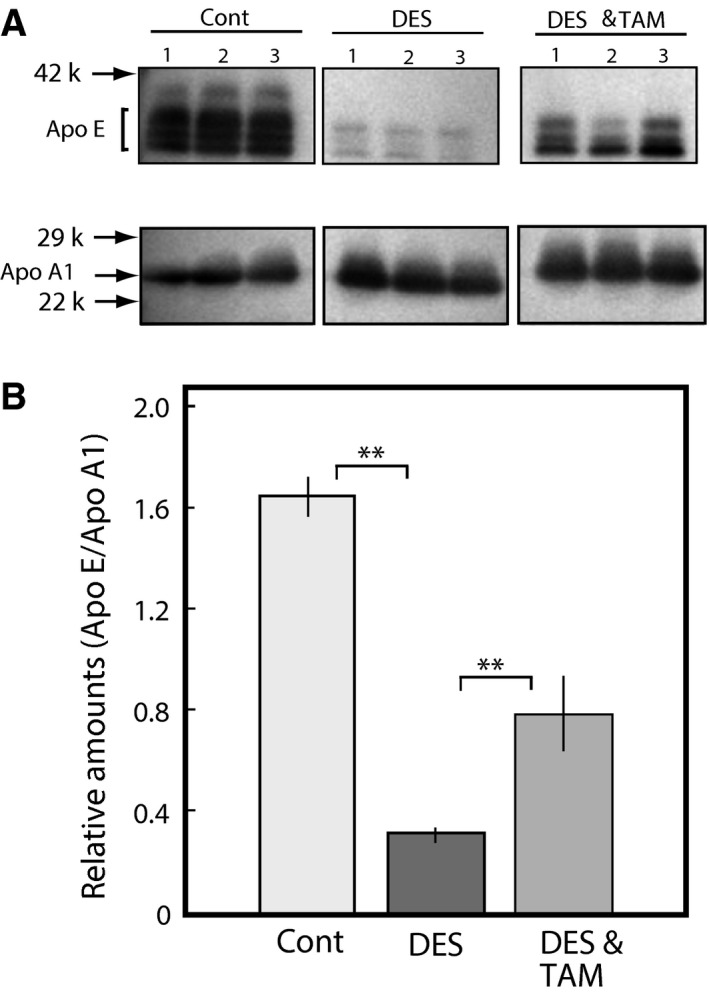
Effects of TAM on the Apo E levels in the serum of adult male rats administered with DES. DES was orally administered at single dose (0.1 mg per rat) (DES), coadministered DES and antiestrogen tamoxifen (DES and TAM) and of control rats administered olive oil only (Cont) as described in the Materials and methods section. The blood was collected at 24 h after the single dose and the serum was prepared as described in the Materials and methods section. Apo E and Apo A1 were analyzed by western blotting using each antibody in the sera (A). The relative levels of Apo E and Apo A1 in each group were determined by densitometrical analysis (B). The data for each group represent the mean ± SEM of three rats. **P < 0.01 compared with each group.

We previously identified the novel adverse effects of a typical endocrine disruptor, diethylstilbestrol, on adrenal steroidogenesis that were dependent on the insufficient supply of HDL cholesterol to the adrenal glands 14. This insufficient supply of HDL cholesterol is caused by a reduction in Apo E secretion from the liver 15. In this study, we found that another synthetic estrogen, ethinylestradiol, also suppressed HDL/cholesterol caused by a delay of Apo E secretion from the liver. Decreased levels of total serum cholesterol and HDL cholesterol caused by DES were partially recovered by coadministration with the antiestrogen, TAM, which binds to the estrogen receptor at same dose (Fig. 1). Serum Apo E reduction by DES was also recovered by coadministration with TAM at same dose as shown in Fig. 2. These results suggest that DES has adverse effects on liver Apo E secretion, leading to disruption of adrenal steroidogenesis after binding to the ER.

### Synthetic estrogens suppressed serum HDL/cholesterol and Apo E

**Figure 3:**
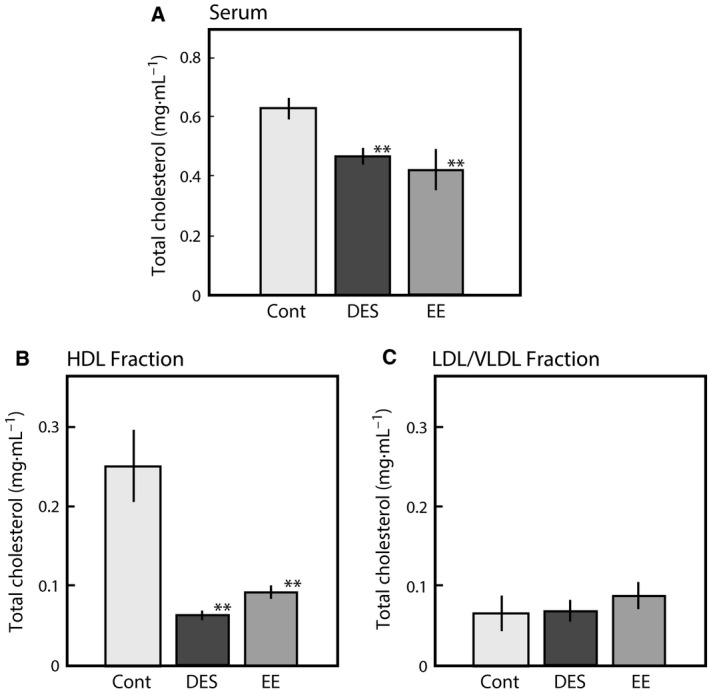
Effects of EE on the total cholesterol levels in the adult female rats. DES and EE were orally administered at single dose to female rats [0.1 (mg DES or EE per rat) per 2 days] as described in the Materials and methods section. The blood was collected at 24 h after the single dose. The amount of total cholesterol in the serum (A), HDL fraction (B), and LDL/VLDL fraction (C) of rats administered DES or EE and of control rats administered olive oil only (Cont) were determined. Cholesterol levels were determined by the enzyme methods using oxidase and cholesterol esterase as described in the Materials and methods section. The data for each group represent the mean ± SEM of three to five rats. **P < 0.01 compared with control rats.

**Figure 4:**
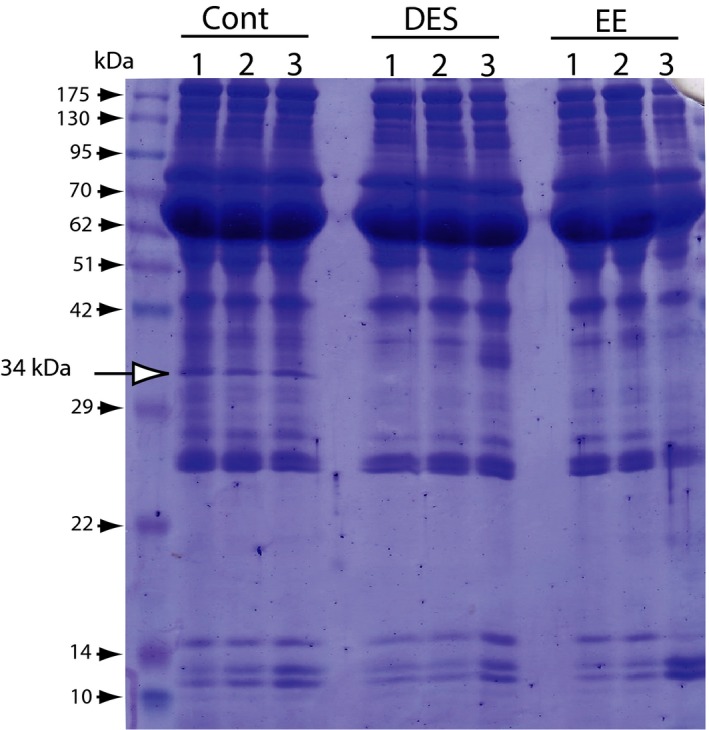
Serum proteins in the rats administered DES and EE. After the single dose of DES and EE (0.1 mg DES or EE per female rat), the blood was collected as described in the Materials and methods section. The serum proteins were diluted 30‐fold with PBS and analyzed by SDS/PAGE. The protein band corresponding to approximately 34 kDa was absent in the sera from the rats administered DES. The protein was identified as Apolipoprotein E (Apo E) by MALDI‐TOF MS analysis method, as described previously 15. Figure reproduced from Ref. 15.

**Figure 5:**
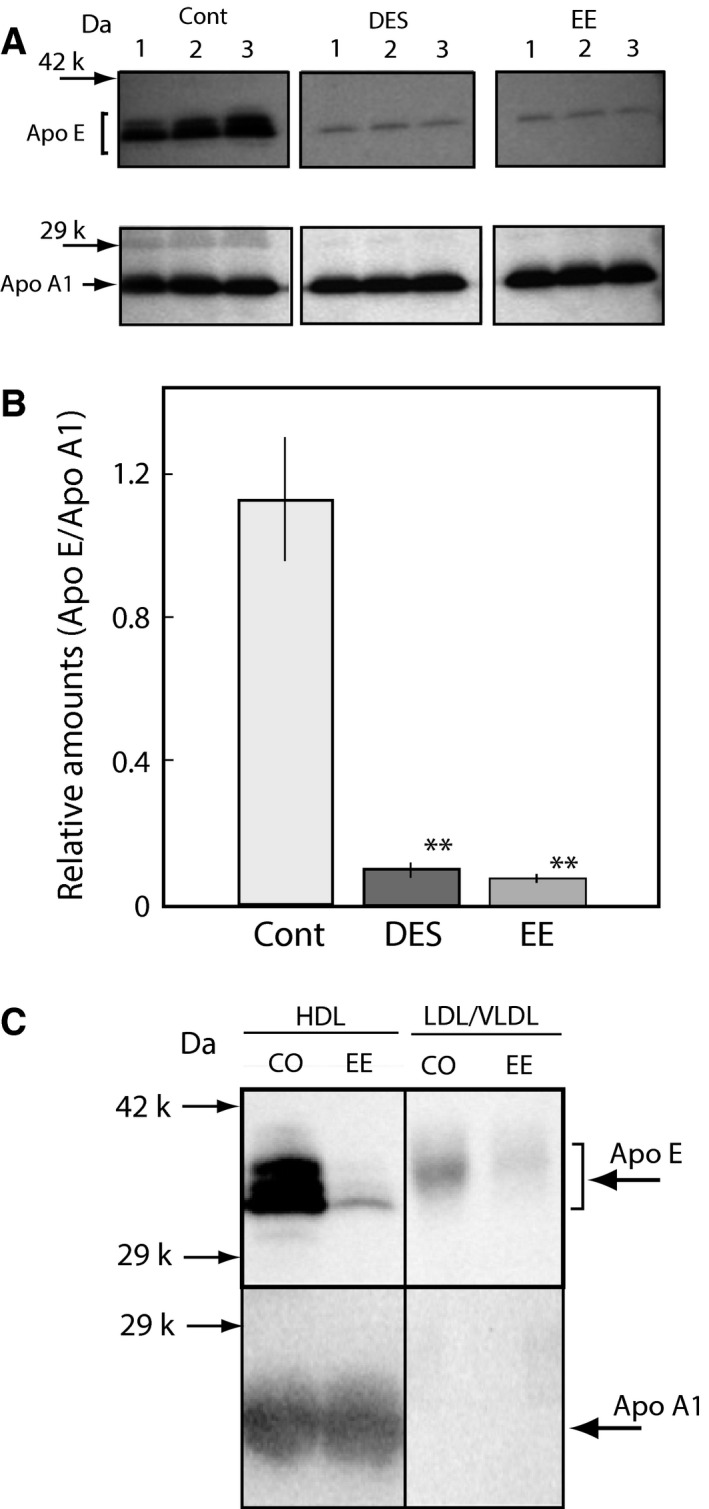
Effects of EE on Apo E levels in the serum of adult female rats. DES and EE were orally administered at single dose to female rats [0.1 (mg DES or EE per rat) per 2 days] as described in the Materials and methods section. The blood was collected at 24 h after the single dose. Apo E and Apo A1 were analyzed by western blotting using each antibody in the sera of each group (A). The relative levels of Apo E and Apo A1 in each group were determined by densitometrical analysis (B). The levels of Apo E and Apo A1 in the HDL fraction and LDL/VLDL fraction of rats administered DES or EE and of control rats administered olive oil only (Cont) were analyzed. Apo A1 was positive only in the LDL/VLDL fraction (C), indicating that the preparation of each lipoprotein fraction was performed successfully. The data for each group in the B panel represent the mean ± SEM of three rats. **P < 0.01 compared with control rats.

**Figure 6:**
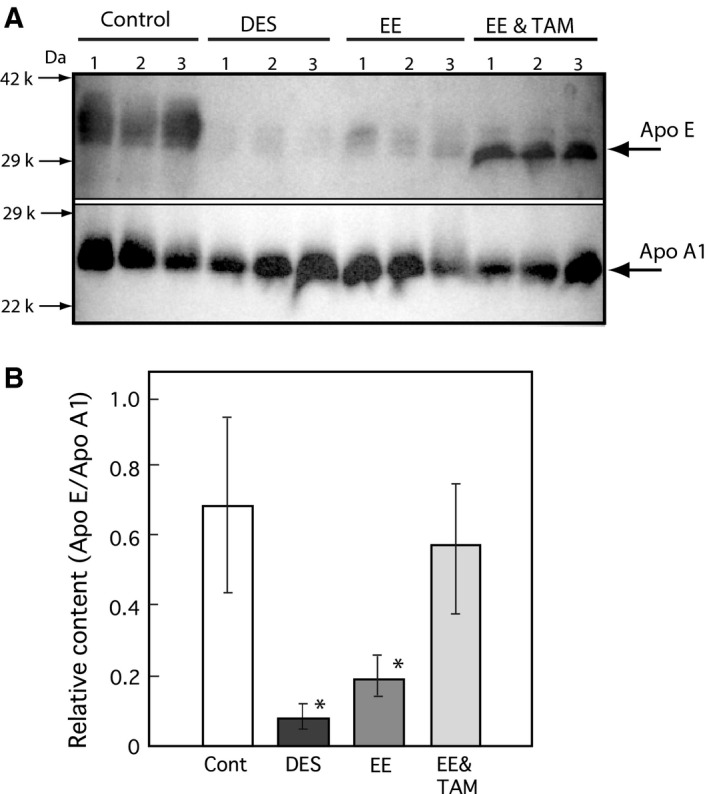
Effects of TAM on Apo E levels in the serum of adult female rats administered with ethinylestradiol. DES and EE were orally administered at single dose to female rats [0.1 (mg DES or EE2 per rat) per 2 days] and both EE and TAM at same dose were coadministered as described in the Materials and methods section. Panel (A) The blood was collected at 24 h after the single dose. Apo E and Apo A1 were analyzed by western blotting using each antibody in the sera of each group (three rats in a group). Control rats were administered olive oil only (Cont). Panel (B) The relative protein expression levels (Apo E/Apo A1) were analyzed densitometrically using a cs analyzer (ATTO) described in the Materials and methods section. The data for each group represent the means ± SEM of three rats. *P < 0.05 compared with controls.

For confirmation of this hypothesis, another synthetic estrogen, EE, which is a main component of oral contractive pills, was administered to female rats, and the effects on cholesterol and Apo E levels were examined. As shown in Fig. 3, EE suppressed the serum cholesterol (A) and HDL cholesterol (B) with only a single dose (0.33 mg per female rat), similar to DES. The protein expression in the serum of rats treated with DES was assessed using SDS/PAGE as shown in Fig. 4. The protein band corresponding to approximately 34 kDa was clearly absent in the serum 12 h after EE‐treatment, similar to the DES‐treated female rats (Fig. 4). The protein was identified as Apo E by MALDI‐TOF mass spectrometry (MS) analysis using a previously described method 15. A specific antibody against rat Apo E reacted with the 34 kDa protein band (Fig. 5A). Although there was no change in Apo A1 expression, the Apo E expression level was clearly decreased in the blood 12 h after EE‐treatment (Fig. 5A,B). Apo E is present as a constituent of chylomicrons, chylomicron remnants, VLDL, intermediate‐density lipoproteins (IDL), LDL, and HDL. Apo E levels were assayed in both fractions of HDL and VLDL/LDL, and the results are presented in Fig. 5C. Decreases in the Apo E levels in the serum and HDL fraction were observed in the female rats administered with EE as shown in Fig. 5. EE was coadministered with TAM at same dose and the results of Apo E expression levels were shown in Fig. 6. As same as that of DES‐treatment, the decrease in Apo E in EE‐treated female rats was restored by coadministration with TAM as shown in Figs 1 and 6.

### Apo E secretion from the liver was suppressed

**Figure 7:**
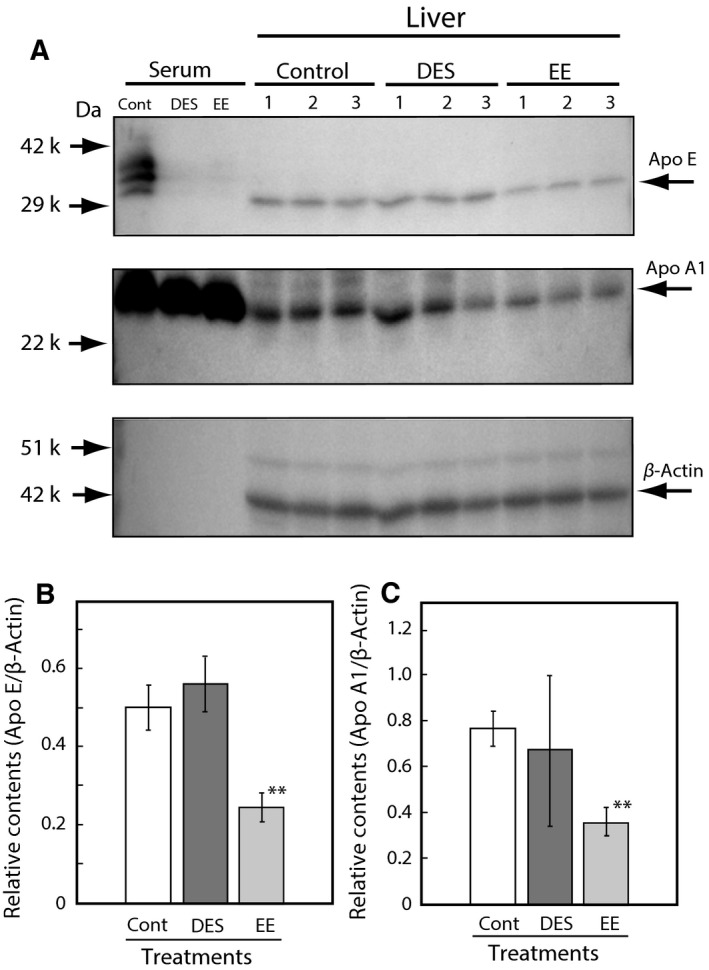
Apo E and Apo A1 expressions in the liver of adult female rats administered with DES and EE. DES and EE were orally administered at single dose to female rats [0.1 (mg DES or EE2 per rat) per 2 days, three rats in a group] as described in the Materials and methods section. Panel (A) The liver was isolated from the rat at 24 h after the single dose. Apo E and Apo A1 in the sera and liver homogenates were analyzed by western blotting using each antibody in each group. Control rats were administered olive oil only (Cont). Panel (B, C) The relative protein expression levels of Apo E/β‐actin and Apo A1/β‐actin were analyzed densitometrically using a cs analyzer (ATTO) described in the Materials and methods section. The data for each group represent the means ± SEM of three rats. **P < 0.01 compared with controls.

**Figure 8:**
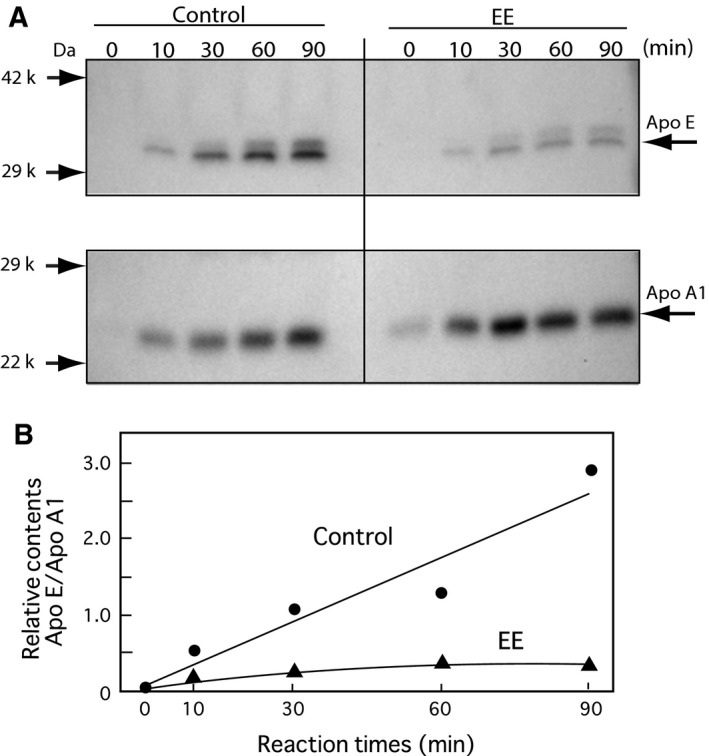
Apo E and Apo A1 secretions levels from the perfused liver. Female rats were treated with a single‐dose EE [(0.1 (mg per rat) per 2 days]. The liver perfusion was performed as described in the Materials and methods section and our previous study 15 for 90 min. Panel (A) The levels of the secreted Apo E and Apo A1 in the perfusates (Final 300 mL) were determined by western blotting analysis using each antibody. The 50‐μL perfusates were electrophoresed for the western blotting analysis using the antibodies against Apo E and Apo A1. Panel (B) The relative protein expression levels (Apo E per Apo A1) were analyzed densitometrically using a cs analyzer (ATTO) described in the Materials and methods section.

Liver expression levels of Apo E, which is mainly expressed and secreted from the liver, are shown in Fig. 7. The expression levels of Apo E protein in DES‐treated rats were not changed (Fig. 7A,B); however, Apo E and Apo A1 in the liver of the EE‐treated rats were slightly decreased compared to the β‐actin expression levels (Fig. 7A,B). Finally, the secretion of Apo E from the liver of rats was analyzed using a liver perfusion experiment as previously shown for DES‐treated rats 15. The Apo E levels in the perfusion liquids were assessed by immunoblotting analysis and the results are shown in Fig. 8. Apo E secretion from the liver compared to Apo A1 was suppressed in the female rats administered with EE (Fig. 8).

## Discussion

There are many toxicities of the reproductive and endocrine systems caused by DES; however, the molecular mechanisms of DES toxicity remains unclear. In previous reports, the adverse effects by DES were caused by direct damage to the reproductive and endocrine systems 22, 23. Recently, we found the indirect adverse effects of DES on the adrenal steroidogenesis via liver dysfunction 14, 15. In this study, we newly found that another synthetic estrogen, EE, also had the same adverse effects as DES.

### Adverse effects of DES via ER

The decreases in serum HDL cholesterol and in Apo E suppression caused by DES exposure were partially recovered by coadministration with the antiestrogen, TAM, suggesting that the adverse effects of DES (0.1 mg per rat) occur after binding to the ER. This indicates that the other synthetic estrogen, EE, may have the adverse activity as DES because EE can bind ER with as high an affinity as DES.

### EE affects cholesterol metabolism

Ethinylestradiol possesses extremely potent estrogen activity similar to DES 24. The exposure to EE at only a single dose suppressed Apo E secretion from the liver, leading to a decrease in HDL cholesterol as shown Figs 3, 4 and 6. EE is a main constituent of oral contraceptives (COCs). The association between venous thromboembolism (VTE) and oral contraceptives that contain estrogen and progestin was observed 25. Thrombotic risk is increased with high‐estrogen COCs relative to standard and low‐dose estrogen COCs. The major COCs prescribed contained 20–35 μg of EE. Estrogen increased the gene expression and plasma levels of coagulation factors 26 and decreased anticoagulation factors, such as protein S 27. In this study, we found that EE has adverse effects on the liver secretion of Apo E, which plays a crucial role in the formation of atherosclerosis through reverse cholesterol transport. Apo E is a 34 kDa glycoprotein produced primarily by hepatocytes and plays a crucial role in atherosclerosis via reverse cholesterol transport after the formation and maturation of HDL particles 28. The suppression of Apo E secretion and the decrease in HDL cholesterol by only a single dose of EE (0.1 mg per rat) suggests that lower but repeated doses of EE may increase the risk of atherosclerosis. These data suggest that the decrease in Apo E secretion from the liver by EE may be a preliminary step for increasing the risk of VTE in women commonly using COCs.

### Estimate of suppressed Apo E secretion

It is interesting to investigate the mechanism of Apo E suppression from the liver by EE exposure. The Apo E expression level in the liver was not significantly suppressed by treatment with DES as shown in Fig. 7. By contrast, the expression levels of Apo E and Apo A1 were suppressed in the liver by exposure to EE (Fig. 7). The secretion rates of Apo A1 was not decreased significantly from the liver, but the rate of only Apo E was suppressed in the liver from rats administered EE (Fig. 8). These results suggest that the Apo E secretion system may be inhibited by any unknown mechanism. Kurano *et al*. 29 reported that treatment with TO901317, an LXR (Liver X‐receptor) agonist, significantly increased Apo E protein secretion from HepG2 cells, which was also associated with the increased expression of Apo E mRNA. A preliminary communication from the coauthors suggests that DES did not change the hepatic expression of LXR, but that it may affect LXR function via endogenous ligands, such as oxysterol. EE may also disrupt the regulation of LXR expression and/or function. For further understanding of the mechanism of EE effects, it will be useful to investigate the effects on male rats.

## Conclusions

It is concluded that these synthetic estrogens discussed in this paper suppressed HDL/cholesterol caused by delayed Apo E secretion from the liver, and this may possibly lead to not only endocrine disruption via a decrease in adrenal steroidogenesis but also an increase in the risk of arterial diseases, such as atheromas.

## Author contributions

HY conceived and supervised the study; HY, KY, and HI designed experiments; KY, NM, and MI performed experiments; HY wrote the manuscript.
